# Supplementary material for: Three-Dimensional Organization of Chicken Genome Provides Insights into Genetic Adaptation to Extreme Environments
Source: Genes (Basel). 2022 Dec 9;13(12):2317. doi: 10.3390/genes13122317 (PMC9778438; doi:10.3390/genes13122317)
Supplement: Supplementary file 1 [file genes-13-02317-s001.zip › genes-2002664-supplementary figures.pdf]

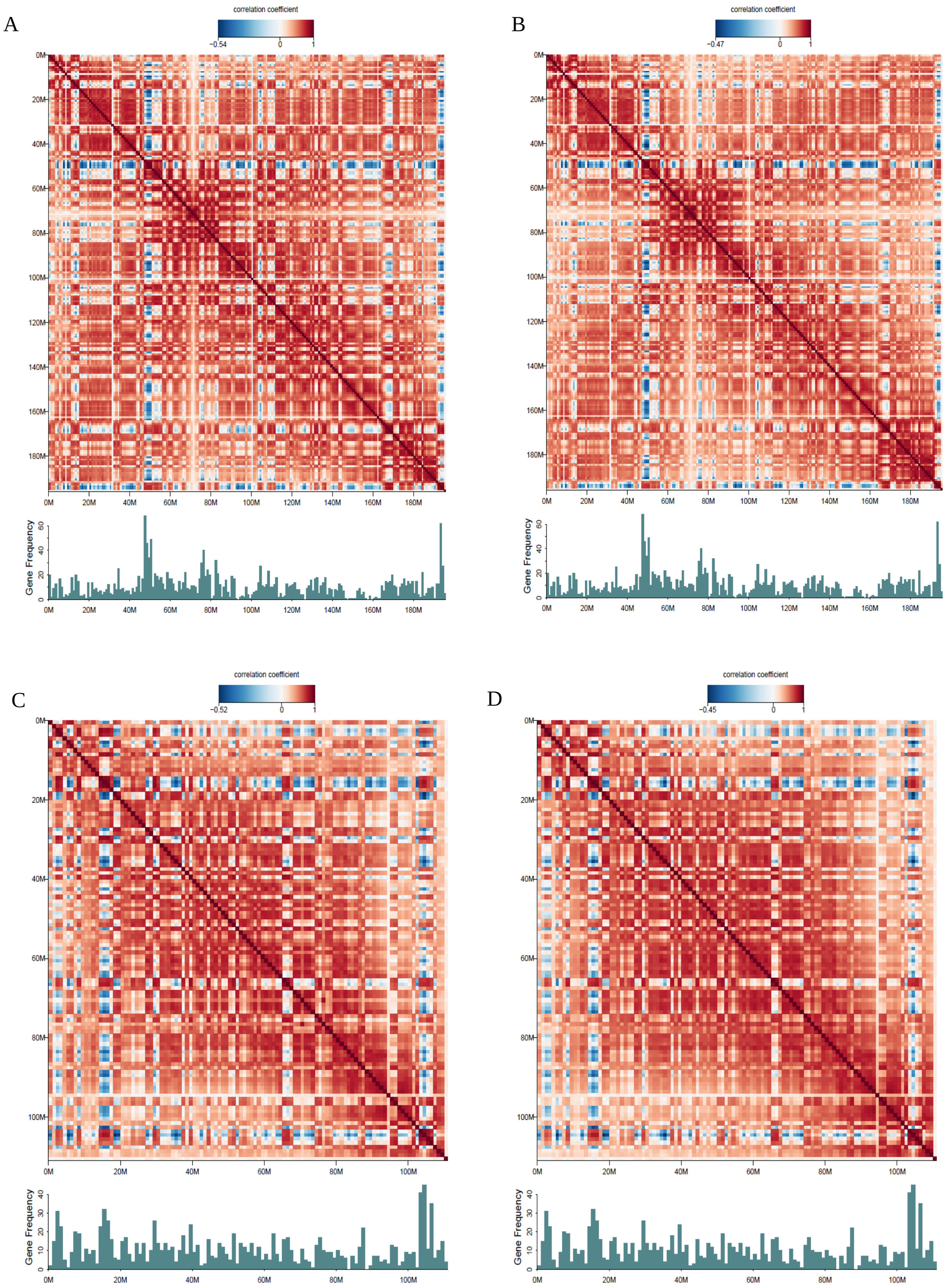
A
correlation coefficient
−0.54
0
1
0M
20M
40M
60M
80M
100M
120M
140M
160M
180M
Gene Frequency
0
20
40
60
B
correlation coefficient
−0.47
0
1
C
correlation coefficient
−0.52
0
1
0M
20M
40M
60M
80M
100M
Gene Frequency
0
10
20
30
40
D
correlation coefficient
−0.45
0
1

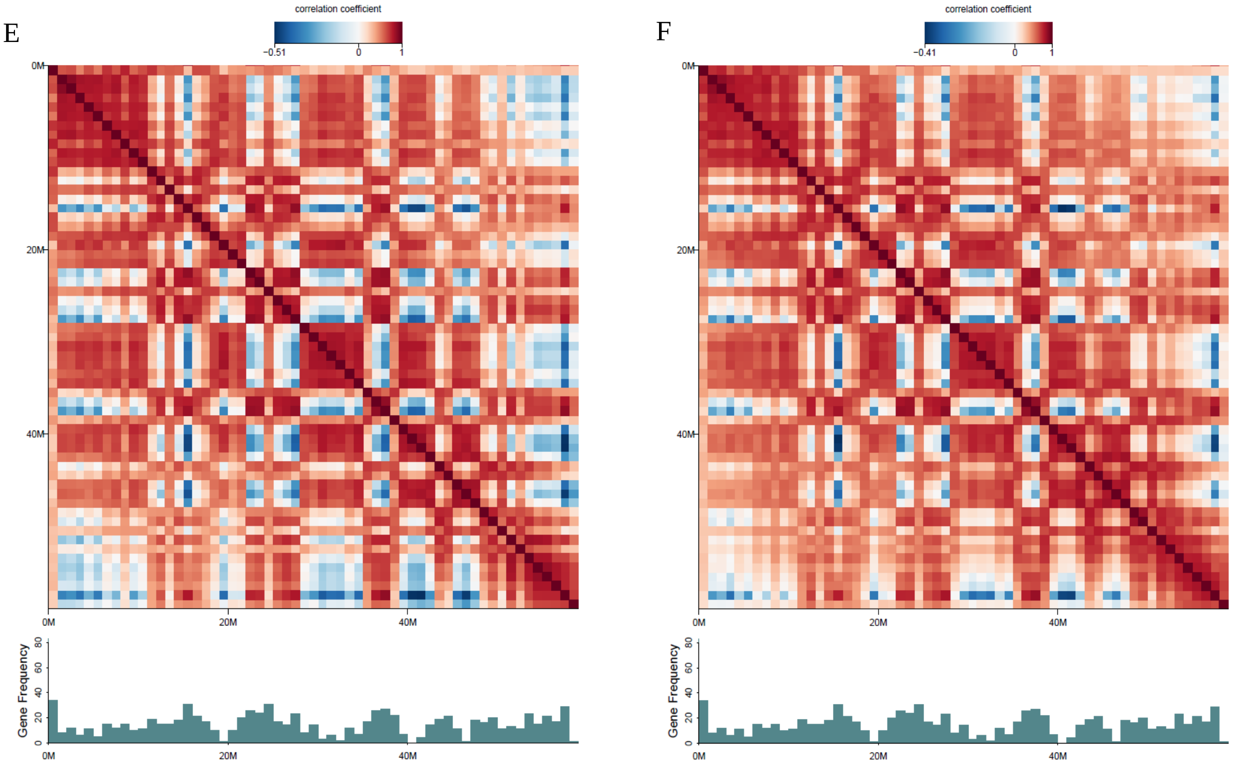

Supplementary Figure S1. Chromatin interaction heatmaps of hepatocytes in LDC (A, C, E) and WCC (B, D, F) on chromosome 1, 3, and 5, respectively. The color of each dot on heatmaps represents the log of the interaction probability for the corresponding pair of genomic loci according to standard JuiceBox color scheme.

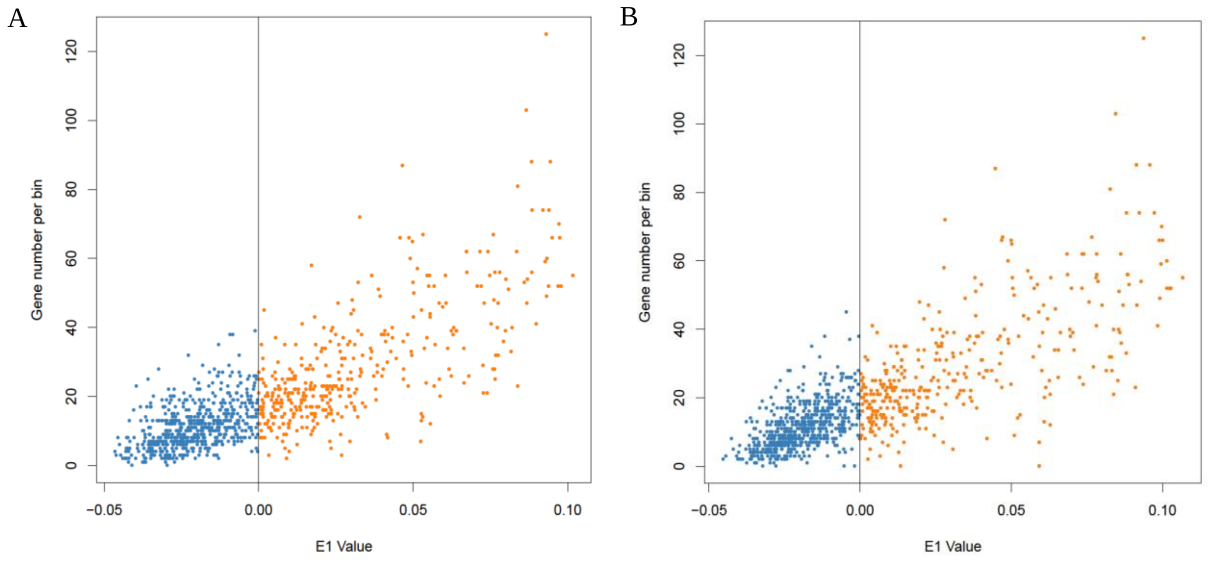

Supplementary Figure S2. Gene number scatter diagram of Compartments A/B in LDC (A) and WCC(B) whole genome. The E1 Value of genes greater than 0 were considered as A Compartments (Orange point), or were B Compartments (Blue point).

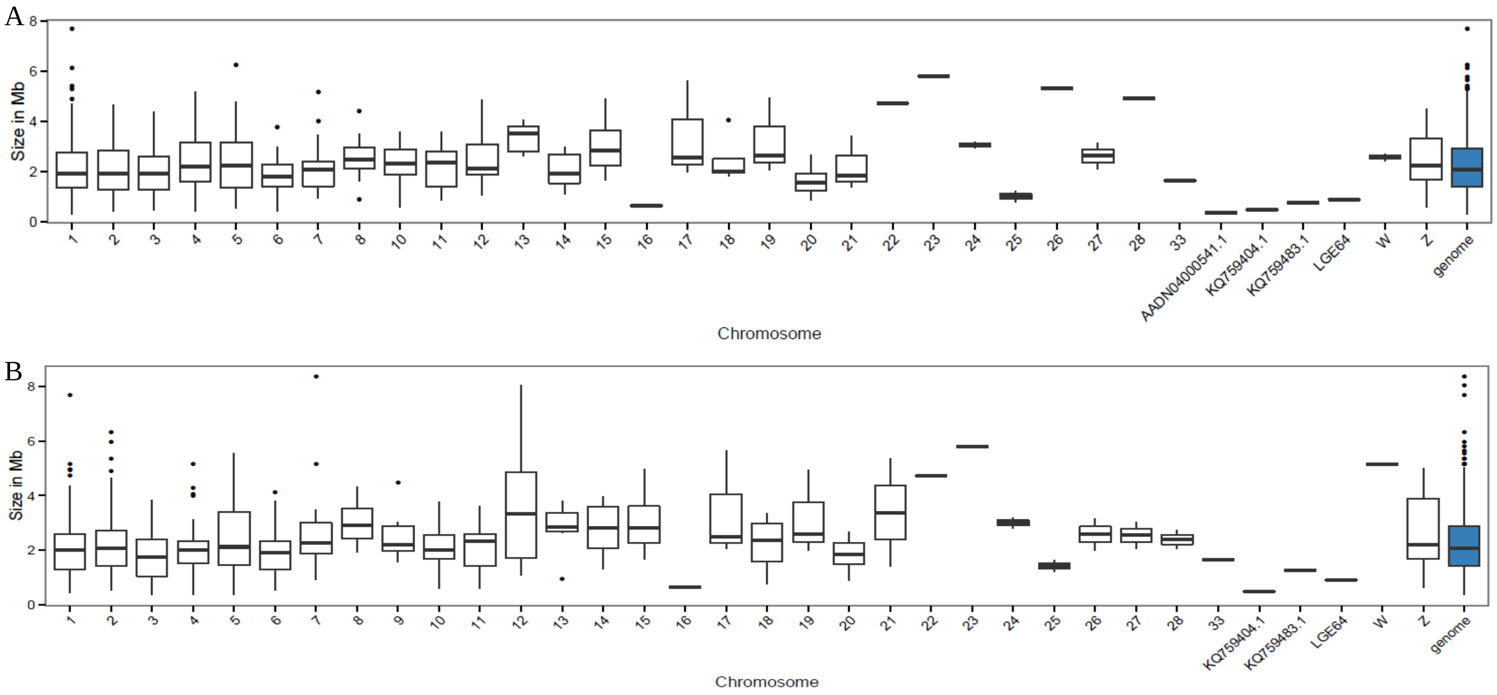

Supplementary Figure S3. The distribution graph of TAD length in LDC(A) and WCC(B) whole genome.

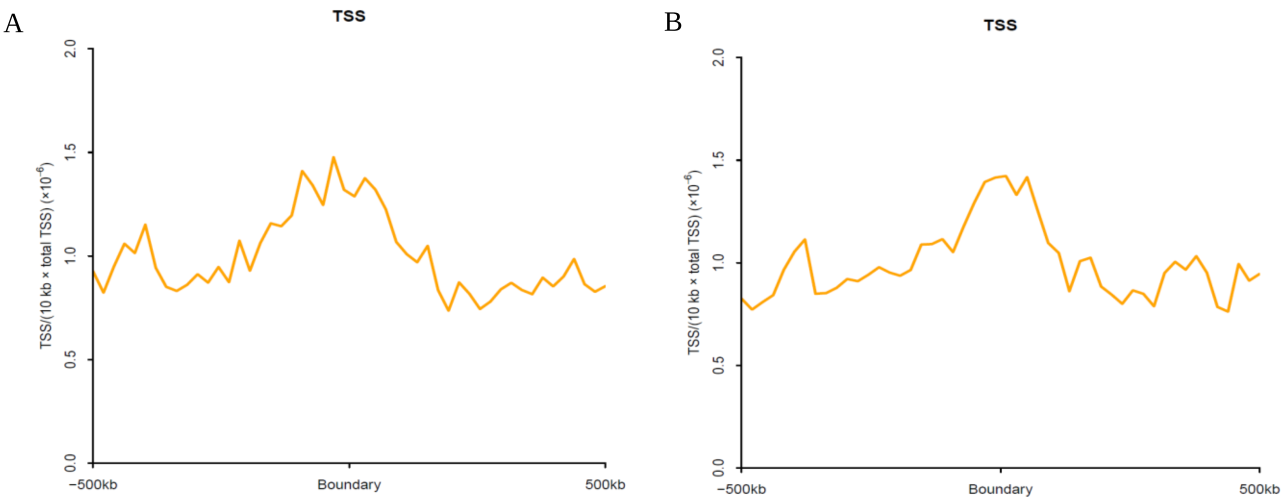

Supplementary Figure S4. The transcription start site (TSS) enrichment in TAD boundary of LDC (A) and WCC (B) whole genome.
