# Supplementary material for: Three-Dimensional Organization of Chicken Genome Provides Insights into Genetic Adaptation to Extreme Environments
Source: Genes (Basel). 2022 Dec 9;13(12):2317. doi: 10.3390/genes13122317 (PMC9778438; doi:10.3390/genes13122317)
Supplement: Supplementary file 1 [file genes-13-02317-s001.zip › genes-2002664-supplementary tables.pdf]

Supplementary Tables

Supplementary Table S1. Primary results of Hi-C libraries sequencing

Sample	Raw Base	Clean Base	Effective Rate	Error Rate	Q20	Q30	GC Content
	bp	bp	%	%	%	%	%
LDC	151,312,880,100	150.325,686,900	99.35	0.04	97.00	92.32	43.81
WCC	145,779,275,400	144,463,788,300	99.10	0.04	96.44	91.12	44.12

Supplementary Table S2. Mapping results of Hi-C sequencing data

Sample	Number of all reads	Number of unique read	intra-chromosomal	Inter-chromosomal	Effective rate
	bp	bp	reads bp	reads bp	%
LDC	321,390,644	262,260,125	207,914,357	54,345,768	83.95
WCC	306,349,326	263,754,929	206,471,795	57,283,134	86.10
Average	313,869,985	263,007,527	207,193,076	55,814,451	85.03

Supplementary Table S3. Identification of TADs in LDC and WCC genome

Sample	Number	Length.average	Length.median
LDC	396	2260019.937	2080001
WCC	386	2336757.269	2080001

Supplementary Table S4. Identification of topological boundary-like regions in LDC and WCC genome.

Chr	start	end	Sample name	Chr	start	end	Sample name
1	7680000	7760000	WCC	1	4080000	4120000	LDC
1	10000000	10280000	WCC	1	7640000	7680000	LDC
1	12360000	12440000	WCC	1	10000000	10280000	LDC
1	15920000	16040000	WCC	1	13680000	13760000	LDC
1	18680000	18720000	WCC	1	15720000	16000000	LDC
1	19960000	20360000	WCC	1	18720000	18720000	LDC
1	20800000	20960000	WCC	1	20080000	20400000	LDC
1	25280000	25360000	WCC	1	25280000	25360000	LDC
1	26400000	26480000	WCC	1	26440000	26480000	LDC
1	32880000	33000000	WCC	1	32920000	33000000	LDC
1	41480000	41840000	WCC	1	41520000	41720000	LDC
1	43120000	43160000	WCC	1	43120000	43160000	LDC
1	43920000	44120000	WCC	1	43880000	44120000	LDC
1	45920000	46040000	WCC	1	47760000	47960000	LDC
1	47760000	47920000	WCC	1	49040000	49440000	LDC
1	49040000	49200000	WCC	1	54280000	54360000	LDC
1	55560000	55800000	WCC	1	55600000	55800000	LDC
1	57000000	57080000	WCC	1	57000000	57080000	LDC
1	59280000	59600000	WCC	1	59280000	59640000	LDC
1	62200000	62280000	WCC	1	62200000	62240000	LDC
1	67000000	67240000	WCC	1	66920000	67240000	LDC
1	68600000	68640000	WCC	1	68560000	68600000	LDC
1	71080000	71320000	WCC	1	70160000	70280000	LDC
1	72320000	72360000	WCC	1	71040000	71320000	LDC
1	77640000	78000000	WCC	1	72320000	72320000	LDC
1	79760000	79880000	WCC	1	73480000	73560000	LDC
1	80640000	80760000	WCC	1	75160000	75520000	LDC
1	82400000	82440000	WCC	1	77720000	77840000	LDC
1	83000000	83160000	WCC	1	80600000	80760000	LDC
1	84960000	85000000	WCC	1	83160000	83400000	LDC
1	87160000	87440000	WCC	1	84960000	84960000	LDC
1	88840000	88920000	WCC	1	87160000	87440000	LDC
1	91160000	91200000	WCC	1	88840000	88880000	LDC
1	92920000	93000000	WCC	1	91400000	91800000	LDC
1	96040000	96280000	WCC	1	96080000	96280000	LDC
1	97480000	97600000	WCC	1	96600000	96720000	LDC
1	99440000	99560000	WCC	1	99440000	99560000	LDC
1	102440000	102480000	WCC	1	1.02E+08	1.02E+08	LDC
1	103960000	104000000	WCC	1	1.04E+08	1.04E+08	LDC
1	104520000	104680000	WCC	1	1.05E+08	1.05E+08	LDC
1	105840000	105960000	WCC	1	1.06E+08	1.06E+08	LDC
1	106640000	106680000	WCC	1	1.07E+08	1.07E+08	LDC

Chr	start	end	Sample name	Chr	start	end	Sample name
1	108840000	109080000	WCC	1	1.09E+08	1.09E+08	LDC
1	112960000	113360000	WCC	1	1.13E+08	1.13E+08	LDC
1	117720000	118040000	WCC	1	1.18E+08	1.18E+08	LDC
1	119640000	119800000	WCC	1	1.2E+08	1.2E+08	LDC
1	125680000	125880000	WCC	1	1.28E+08	1.29E+08	LDC
1	128360000	128480000	WCC	1	1.34E+08	1.34E+08	LDC
1	134120000	134200000	WCC	1	1.36E+08	1.36E+08	LDC
1	136200000	136400000	WCC	1	1.44E+08	1.44E+08	LDC
1	143760000	143840000	WCC	1	1.46E+08	1.46E+08	LDC
1	146280000	146520000	WCC	1	1.52E+08	1.53E+08	LDC
1	156280000	156520000	WCC	1	1.56E+08	1.56E+08	LDC
1	161680000	161840000	WCC	1	1.62E+08	1.62E+08	LDC
1	165600000	165680000	WCC	1	1.66E+08	1.66E+08	LDC
1	171320000	171480000	WCC	1	1.7E+08	1.71E+08	LDC
1	173760000	173880000	WCC	1	1.71E+08	1.71E+08	LDC
1	181160000	181320000	WCC	1	1.74E+08	1.74E+08	LDC
1	182840000	183120000	WCC	1	1.81E+08	1.81E+08	LDC
1	184800000	184960000	WCC	1	1.83E+08	1.83E+08	LDC
1	192640000	192920000	WCC	1	1.85E+08	1.85E+08	LDC
2	5320000	5360000	WCC	1	1.93E+08	1.93E+08	LDC
2	12840000	13040000	WCC	2	5320000	5360000	LDC
2	18360000	18560000	WCC	2	8480000	8640000	LDC
2	25080000	25400000	WCC	2	12880000	13040000	LDC
2	27760000	28160000	WCC	2	14000000	14120000	LDC
2	30880000	31160000	WCC	2	18360000	18400000	LDC
2	35520000	35560000	WCC	2	22400000	22560000	LDC
2	40200000	40280000	WCC	2	25080000	25120000	LDC
2	44840000	45000000	WCC	2	26280000	26320000	LDC
2	49880000	50040000	WCC	2	30960000	31160000	LDC
2	60400000	60440000	WCC	2	35480000	35520000	LDC
2	62440000	62480000	WCC	2	40200000	40280000	LDC
2	64480000	64720000	WCC	2	42480000	42880000	LDC
2	68880000	69120000	WCC	2	44800000	45000000	LDC
2	75080000	75160000	WCC	2	49920000	50080000	LDC
2	81520000	81640000	WCC	2	52880000	53240000	LDC
2	83760000	84000000	WCC	2	54360000	54400000	LDC
2	86040000	86160000	WCC	2	56680000	57080000	LDC
2	89160000	89200000	WCC	2	57920000	58240000	LDC
2	91160000	91200000	WCC	2	60400000	60520000	LDC
2	92400000	92560000	WCC	2	62440000	62600000	LDC
2	96600000	96720000	WCC	2	63360000	63400000	LDC
2	98920000	99160000	WCC	2	64520000	64720000	LDC
2	100880000	100960000	WCC	2	66520000	66840000	LDC

Chr	start	end	Sample name	Chr	start	end	Sample name
2	102080000	102440000	WCC	2	68840000	68920000	LDC
2	103400000	103720000	WCC	2	75120000	75200000	LDC
2	114360000	114720000	WCC	2	81520000	81560000	LDC
2	121920000	122160000	WCC	2	84480000	84520000	LDC
2	126400000	126640000	WCC	2	86000000	86120000	LDC
2	130000000	130080000	WCC	2	89200000	89400000	LDC
2	132760000	132840000	WCC	2	92320000	92440000	LDC
2	135800000	135840000	WCC	2	96600000	96720000	LDC
2	136360000	136760000	WCC	2	98920000	99160000	LDC
2	139440000	139480000	WCC	2	1.02E+08	1.02E+08	LDC
2	142840000	143240000	WCC	2	1.03E+08	1.04E+08	LDC
3	4600000	4680000	WCC	2	1.06E+08	1.06E+08	LDC
3	5680000	5720000	WCC	2	1.08E+08	1.08E+08	LDC
3	7640000	7680000	WCC	2	1.11E+08	1.11E+08	LDC
3	8520000	8760000	WCC	2	1.19E+08	1.19E+08	LDC
3	10040000	10160000	WCC	2	1.22E+08	1.22E+08	LDC
3	15320000	15360000	WCC	2	1.26E+08	1.27E+08	LDC
3	18080000	18080000	WCC	2	1.3E+08	1.3E+08	LDC
3	18840000	18880000	WCC	2	1.36E+08	1.36E+08	LDC
3	21040000	21080000	WCC	2	1.43E+08	1.43E+08	LDC
3	25920000	26240000	WCC	3	4560000	4640000	LDC
3	27880000	28000000	WCC	3	5720000	5720000	LDC
3	35400000	35440000	WCC	3	7680000	7680000	LDC
3	37640000	37720000	WCC	3	8640000	8800000	LDC
3	40800000	40840000	WCC	3	10000000	10120000	LDC
3	41200000	41320000	WCC	3	15320000	15360000	LDC
3	43080000	43440000	WCC	3	18840000	18840000	LDC
3	46360000	46440000	WCC	3	21040000	21200000	LDC
3	52960000	53200000	WCC	3	25920000	26240000	LDC
3	54520000	54600000	WCC	3	27880000	27960000	LDC
3	56560000	56600000	WCC	3	33680000	34000000	LDC
3	60440000	60520000	WCC	3	34880000	34920000	LDC
3	62920000	63160000	WCC	3	37680000	37720000	LDC
3	65960000	66000000	WCC	3	40120000	40240000	LDC
3	68280000	68320000	WCC	3	42560000	42680000	LDC
3	69000000	69080000	WCC	3	46320000	46400000	LDC
3	75240000	75320000	WCC	3	52960000	53200000	LDC
3	79920000	80000000	WCC	3	54560000	54680000	LDC
3	85840000	85880000	WCC	3	56560000	56600000	LDC
3	86960000	87080000	WCC	3	60400000	60520000	LDC
3	89080000	89480000	WCC	3	62960000	63160000	LDC
3	91040000	91160000	WCC	3	64040000	64280000	LDC
3	104440000	104480000	WCC	3	65960000	66120000	LDC

Chr	start	end	Sample name	Chr	start	end	Sample name
3	107880000	108040000	WCC	3	69000000	69040000	LDC
4	2120000	2440000	WCC	3	75320000	75360000	LDC
4	4360000	4520000	WCC	3	79880000	79920000	LDC
4	5200000	5280000	WCC	3	85840000	85880000	LDC
4	6000000	6160000	WCC	3	87040000	87120000	LDC
4	14400000	14440000	WCC	3	89080000	89280000	LDC
4	16400000	16440000	WCC	3	91080000	91160000	LDC
4	29400000	29440000	WCC	3	98480000	98520000	LDC
4	31280000	31600000	WCC	3	1.04E+08	1.04E+08	LDC
4	33080000	33480000	WCC	3	1.08E+08	1.08E+08	LDC
4	35520000	35600000	WCC	4	2120000	2440000	LDC
4	37840000	37880000	WCC	4	4440000	4520000	LDC
4	47360000	47360000	WCC	4	5200000	5240000	LDC
4	49480000	49520000	WCC	4	6000000	6160000	LDC
4	51280000	51360000	WCC	4	14320000	14400000	LDC
4	55360000	55440000	WCC	4	16440000	16440000	LDC
4	61080000	61400000	WCC	4	29440000	29440000	LDC
4	61760000	61840000	WCC	4	31280000	31600000	LDC
4	66960000	67080000	WCC	4	35480000	35560000	LDC
4	70360000	70440000	WCC	4	37840000	37920000	LDC
4	76120000	76240000	WCC	4	43320000	43440000	LDC
4	82440000	82720000	WCC	4	43880000	43960000	LDC
4	86760000	86800000	WCC	4	45000000	45040000	LDC
4	88920000	88960000	WCC	4	47320000	47360000	LDC
5	7160000	7280000	WCC	4	49520000	49560000	LDC
5	12320000	12480000	WCC	4	51320000	51360000	LDC
5	15640000	15720000	WCC	4	55360000	55400000	LDC
5	17000000	17120000	WCC	4	57320000	57440000	LDC
5	19480000	19520000	WCC	4	61760000	61840000	LDC
5	21400000	21440000	WCC	4	65080000	65360000	LDC
5	23040000	23120000	WCC	4	66960000	67040000	LDC
5	24520000	24800000	WCC	4	70360000	70440000	LDC
5	29520000	29520000	WCC	4	73560000	73560000	LDC
5	33320000	33520000	WCC	4	75000000	75360000	LDC
5	37000000	37000000	WCC	4	76240000	76360000	LDC
5	39400000	39440000	WCC	4	82440000	82720000	LDC
5	41040000	41040000	WCC	4	86760000	86760000	LDC
5	42920000	43080000	WCC	4	88960000	89040000	LDC
5	46160000	46200000	WCC	5	7200000	7360000	LDC
5	46560000	46640000	WCC	5	8720000	8960000	LDC
5	50640000	50720000	WCC	5	12280000	12520000	LDC
6	7680000	7760000	WCC	5	15640000	15720000	LDC
6	9040000	9240000	WCC	5	17000000	17120000	LDC

Chr	start	end	Sample name	Chr	start	end	Sample name
6	10240000	10360000	WCC	5	19480000	19520000	LDC
6	12280000	12640000	WCC	5	21400000	21440000	LDC
6	14760000	14800000	WCC	5	23080000	23120000	LDC
6	16960000	17000000	WCC	5	28120000	28320000	LDC
6	17800000	17840000	WCC	5	33360000	33520000	LDC
6	19120000	19440000	WCC	5	35960000	36120000	LDC
6	19960000	20080000	WCC	5	39360000	39440000	LDC
6	21960000	22080000	WCC	5	41000000	41040000	LDC
6	23640000	23680000	WCC	5	43280000	43400000	LDC
6	25600000	25720000	WCC	5	45160000	45240000	LDC
6	28040000	28080000	WCC	5	46080000	46160000	LDC
7	5160000	5240000	WCC	5	53360000	53560000	LDC
7	10000000	10080000	WCC	6	2760000	3000000	LDC
7	11000000	11040000	WCC	6	7760000	7760000	LDC
7	13880000	13920000	WCC	6	10240000	10360000	LDC
7	16680000	16960000	WCC	6	12080000	12400000	LDC
7	18080000	18280000	WCC	6	14800000	14800000	LDC
7	21760000	21880000	WCC	6	17800000	17840000	LDC
7	23760000	23880000	WCC	6	19160000	19400000	LDC
7	26000000	26120000	WCC	6	19960000	20200000	LDC
7	28520000	28600000	WCC	6	22000000	22120000	LDC
8	3520000	3680000	WCC	6	23640000	23800000	LDC
8	8000000	8080000	WCC	6	25680000	25840000	LDC
8	19640000	19680000	WCC	6	27880000	28040000	LDC
8	21720000	22040000	WCC	6	28800000	29200000	LDC
8	23960000	24120000	WCC	6	30600000	30680000	LDC
8	26720000	27040000	WCC	7	5160000	5280000	LDC
9	3400000	3560000	WCC	7	10040000	10040000	LDC
9	5600000	5720000	WCC	7	12480000	12640000	LDC
9	7920000	8000000	WCC	7	13880000	13920000	LDC
9	12480000	12720000	WCC	7	15520000	15720000	LDC
9	14280000	14320000	WCC	7	16680000	16960000	LDC
9	16280000	16440000	WCC	7	18080000	18280000	LDC
9	18960000	19120000	WCC	7	21760000	21960000	LDC
9	22160000	22200000	WCC	7	23640000	23800000	LDC
10	4240000	4480000	WCC	7	26080000	26120000	LDC
10	6600000	6800000	WCC	7	28520000	28560000	LDC
10	12000000	12240000	WCC	7	32840000	32920000	LDC
10	12840000	13160000	WCC	8	3520000	3600000	LDC
10	14960000	15320000	WCC	8	8000000	8080000	LDC
10	16640000	16920000	WCC	8	14120000	14280000	LDC
11	10000000	10080000	WCC	8	16840000	16840000	LDC
11	11640000	11720000	WCC	8	19640000	19680000	LDC

Chr	start	end	Sample name	Chr	start	end	Sample name
11	14040000	14240000	WCC	8	22000000	22040000	LDC
11	17840000	17880000	WCC	8	24000000	24200000	LDC
12	3400000	3560000	WCC	8	25840000	25880000	LDC
12	8400000	8680000	WCC	8	26760000	26840000	LDC
12	10400000	10800000	WCC	10	4080000	4440000	LDC
12	11880000	11920000	WCC	10	6640000	6760000	LDC
13	4720000	4840000	WCC	10	9000000	9400000	LDC
13	7760000	7800000	WCC	10	12080000	12240000	LDC
13	10440000	10640000	WCC	10	12840000	13160000	LDC
14	9080000	9160000	WCC	10	14080000	14240000	LDC
14	11960000	12000000	WCC	10	16640000	16920000	LDC
15	2440000	2840000	WCC	11	10000000	10080000	LDC
15	4520000	4520000	WCC	11	11640000	11720000	LDC
15	9480000	9600000	WCC	11	14120000	14240000	LDC
17	5080000	5320000	WCC	11	17840000	17880000	LDC
18	3360000	3400000	WCC	12	3400000	3520000	LDC
18	9200000	9200000	WCC	12	8400000	8640000	LDC
19	2600000	2800000	WCC	12	10480000	10800000	LDC
19	4800000	5040000	WCC	12	11880000	11920000	LDC
20	1440000	1760000	WCC	12	16920000	17120000	LDC
20	3760000	3880000	WCC	13	3480000	3720000	LDC
20	5720000	5760000	WCC	13	7800000	7800000	LDC
20	6640000	7000000	WCC	13	10440000	10600000	LDC
20	9520000	9720000	WCC	14	8880000	9120000	LDC
20	11240000	11440000	WCC	14	12000000	12040000	LDC
21	1400000	1520000	WCC	15	2440000	2840000	LDC
25	1640000	1680000	WCC	15	4520000	4520000	LDC
26	2000000	2160000	WCC	15	9440000	9520000	LDC
28	2080000	2240000	WCC	17	5120000	5320000	LDC
Z	2920000	3120000	WCC	18	2000000	2280000	LDC
Z	7080000	7080000	WCC	18	9200000	9240000	LDC
Z	15720000	15920000	WCC	19	2640000	2800000	LDC
Z	18120000	18440000	WCC	19	4880000	5040000	LDC
Z	20560000	20600000	WCC	20	1560000	1640000	LDC
Z	25600000	25680000	WCC	20	3760000	3880000	LDC
Z	27160000	27400000	WCC	20	5760000	5760000	LDC
Z	28200000	28400000	WCC	20	6640000	6920000	LDC
Z	30760000	31000000	WCC	20	7880000	8120000	LDC
Z	39800000	40000000	WCC	20	9480000	9640000	LDC
Z	40640000	40800000	WCC	20	11240000	11440000	LDC
Z	51520000	51600000	WCC	21	1400000	1560000	LDC
Z	56080000	56320000	WCC	21	3400000	3440000	LDC
Z	64960000	65040000	WCC	25	1640000	1680000	LDC

Chr	start	end	Sample name	Chr	start	end	Sample name
Z	67160000	67320000	WCC	Z	2160000	2200000	LDC
Z	70160000	70480000	WCC	Z	2920000	3120000	LDC
Z	73400000	73560000	WCC	Z	7080000	7080000	LDC
Z	78360000	78480000	WCC	Z	13760000	13800000	LDC
				Z	15880000	15920000	LDC
				Z	18200000	18480000	LDC
				Z	20560000	20600000	LDC
				Z	27160000	27400000	LDC
				Z	28120000	28280000	LDC
				Z	30760000	30960000	LDC
				Z	37600000	37920000	LDC
				Z	39800000	39960000	LDC
				Z	40560000	40720000	LDC
				Z	42160000	42360000	LDC
				Z	45640000	45760000	LDC
				Z	47640000	48000000	LDC
				Z	56080000	56320000	LDC
				Z	58920000	59280000	LDC
				Z	64920000	65040000	LDC
				Z	67120000	67320000	LDC
				Z	70360000	70480000	LDC
				Z	73920000	73920000	LDC
				Z	78440000	78480000	LDC
